# Platelet-rich plasma stimulates angiogenesis in mice which may promote hair growth

**DOI:** 10.1186/s40001-017-0278-5

**Published:** 2017-10-11

**Authors:** Hanxiao Cheng, Jufang Zhang, Jinsheng Li, Ming Jia, Yuyan Wang, Haiyan Shen

**Affiliations:** Department of Plastic Surgery, Hangzhou First People’s Hospital, Nanjing Medical University, No. 261 Huansha Road, Hangzhou, 310006 China

**Keywords:** Platelet-rich plasma, Hair growth, Hair follicle

## Abstract

**Background:**

Platelet-rich plasma (PRP) is an autologous concentration of human platelets in plasma. In this paper, we aimed to investigate the effect of PRP on hair growth.

**Methods:**

Platelet-rich plasma and platelet-poor plasma were prepared by sterile centrifugation and injected into shaved dorsal skin of mice (*n* = 10). Saline injection was used in the control group. The length of randomly plucked hairs was measured at 8, 13, 18 days after PRP injection. Histological examination was preformed to observe the histologic changes of skins. The immunohistochemistry analysis of CD31 was performed to detect the changes of hair length and formation of new vessels.

**Results:**

At 13 and 18 days after the last injection, the hair length of mice in PRP group (4.24 ± 0.60 and 8.29 ± 0.48 mm, respectively) was significantly longer compared with the control group (3.70 ± 0.52 and 7.21 ± 0.64 mm, *p* < 0.05). No significant difference in the hair length was found between the PPP group and the control (*p* > 0.05). In addition, the number of CD31-positive vessel in the PRP group (9.90 ± 0.60) was more than that in the control group (8.60 ± 2.34, *p* < 0.05).

**Conclusion:**

Platelet-rich plasma might promote hair length growth and increase the number of hair follicles by inducing angiogenesis.

## Background

Platelet-rich plasma (PRP) is an autologous concentration of human platelets in plasma. Through degranulation of the alpha granules in platelets, PRP can secrete various growth factors, including platelet-derived growth factor (PDGF), vascular endothelial growth factor (VEGF), fibroblast growth factor (FGF), hepatocyte growth factor (HGF), and transforming growth factor (TGF), which have been documented to initiate wound healing [1, 2] and promote the proliferation and transformation of endothelial cells and pericytes into endothelial sprouts [3, 4].

The roles of PRP for the treatment of hair growth have been reported in many recent researches. Uebel et al. [5] have found that platelet plasma growth factors increase the yield of follicular units in male pattern baldness surgery. Recent work has shown that PRP increases the proliferation of dermal papilla cells and induce faster telogen-to-anagen transition using in vivo and in vitro models [6]. Another study has indicated that PRP promotes the hair follicle reconstitution and significantly shorten the time of hair formation [7].

Both the PRP and platelet-poor plasma (PPP) include the full complement of coagulation proteins. In the present study, the influence of PRP and PPP on hair growth in C57BL/6 mice was investigated. The hypothesis was that PRP had a positive effect on hair length growth and increase of the number of hair follicles.

## Methods

### Experimental animals

Totally 50 healthy C57BL/6 male mice (6 weeks old, 20 ± 2 g) were obtained from the Center of Laboratory Animals, Hangzhou Normal University (Hangzhou, China). Animals were fed the same food and maintained in a constant environment under a 12:12-h light–dark cycle. After 1 week of acclimatization, mice were randomly divided into three groups: PRP group (*n* = 10), PPP group (*n* = 10), and control group (*n* = 10).

The study protocol was approved by the institutional ethics committee of animal research under the Law of Animal Research and Statutory Regulations in China.

### Preparations of PRP and PPP

Blood were collected by eyeball removal exsanguinations from 20 adult mice after anesthesia. PRP was prepared using a double-spin method as previously described. Briefly, 15 ml of blood from each mouse was added to a centrifuge tube containing 3.2% (w/v) trisodium citrate (9:1 v/v mixture). The tubes were centrifuged at 800 rpm for 15 min, resulting in three layers: a PPP layer at the top of the tube, a PRP layer in the middle, and an erythrocyte layer at the bottom of the tube. The supernatant yellow plasma (PRP layer) was centrifuged at 3500 rpm for 15 min to concentrate the platelets. Then the platelet-rich pellet was resuspended in 1.0 ml of plasma, and the suspension was collected as PRP. Totally 0.5 M calcium chloride and thrombin (1:1 v/v mixture) was prepared as an activator in advance, and a mixture of PRP and the activator (10:1) was incubated at room temperature for 10 min, yielding activated PRP. The activated PRP was centrifuged at 15,000 rpm for 15 min and stored at − 20 °C until usage. The platelets in PRP and whole blood were microscopically counted.

### Animal experiments

Thirty male mice were randomized into 3 groups: PRP group (*n* = 10), PPP group (*n* = 10), and control group (*n* = 10). At 7 weeks of age, when all hair follicles were in the telogen stage, the dorsum of each mouse was shaved using an animal clipper. PRP and PPP (0.1 ml) were separately injected into the dorsal skin of mice in the PRP and PPP groups by subcutaneous injection, and equivalent volumes of normal saline was injected in the control group for three times at 3-day intervals. Finally, hairs of each mouse were plucked randomly from shaved dorsal area at 8, 13, 18 days after the last injection, and then the average hair length was measured and calculated.

### Hair length measurement

At 8, 13, and 18 days after the last injection, 10 hairs in each mouse were randomly selected in the target area. Hair length measurements were carried out in three fields using an electron microscope, and their average was expressed as millimeters. The elongated or damaged hairs were excluded.

### Hematoxylin and eosin (HE) staining

Dorsal skin samples were excised at 18 days after the third injection. Then samples were fixed in 10% neutral buffered formalin, embedded in paraffin, and cut into 4 μm. The sections were baked for 4 h for deparaffinization at 65 °C, dipped into gradient ethanol, and then stained with hematoxylin for 5 min. After differentiated in 1% hydrochloric acid alcohol, the sections were incubated in ammonia water, stained with eosin, and rinsed with distilled water. Finally, the sections were dehydrated with gradient ethanol, cleared with xylene, mounted with neutral resin, and observed using a light microscopy (Olympus, Tokyo, Japan).

### Immunohistochemistry

Paraffin embedded tissues were sliced in 4 μm and sections were deparaffinized in xylene followed by grated ethanol and rehydrated in PBS (pH 7.5). Then, they were microwaved for 15 min for antigen retrieval and blocked with normal goat serum for 20 min at 37 °C. The sections were incubated overnight at 4 °C with goat anti-mouse CD31 polyclonal antibody (1:50, Santa Cruz, Santa Cruz, USA), followed by washing and then incubated with rabbit anti-goat second antibody (1:50, Maixin Biotech, Fuzhou, China) at 37 °C for 30 min. The sections were stained with 3,3′-diaminobenzidine (DAB, Zhongshan Biotech Co., Ltd, Beijing, China) for 5 min and re-stained with hematoxylin for 2 min. The primary antibody was replaced by PBS in the negative controls. The stained sections were examined by microscope, and three independent fields (250 ×) were randomly selected to calculate the number of positive vessels.

### Statistical analysis

Data are presented as mean ± standard deviations (SD). All statistical analyses were performed using one-way ANOVA followed by a Fisher’s LSD post hoc test by SPSS 17.0 (SPSS Inc., Chicago, IL, USA). A *p* < 0.05 was regarded to be statistically significant.

## Results

### Concentration of platelets in PRP

The mean concentration of platelets in PRP was higher (6.5 × 10^9^ cells/ml) than that in whole blood (0.8 × 10^9^ cells/ml, *p* < 0.05). Then concentration of platelets in PRP was diluted to 4 × 10^9^ cells/ml before injection.

### PRP injection stimulates hair growth

**Fig. 1:**
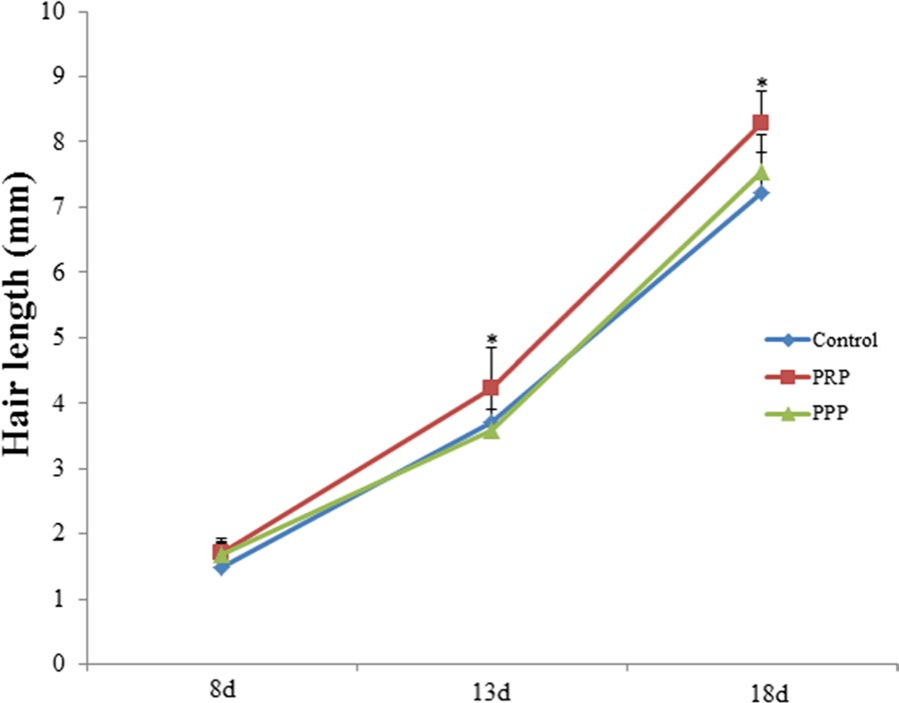
Hair length at 8, 13, 18 days after injection of platelet-rich plasma. *p < 0.05 vs. control group

After analyzing the hair length of mice injected with PRP, PPP, and saline, it was clearly seen that at the 13 and 18 days of the last injection, the hair length of mice in PRP group (4.24 ± 0.60 and 8.29 ± 0.48 mm, respectively) was significantly longer compared with the control group (3.70 ± 0.52 and 7.21 ± 0.64 mm, *p* < 0.05, Fig. 1). However, no significant difference in the hair length was found between the PPP group and the control group at the three detected times (*p* > 0.05).

**Fig. 2:**
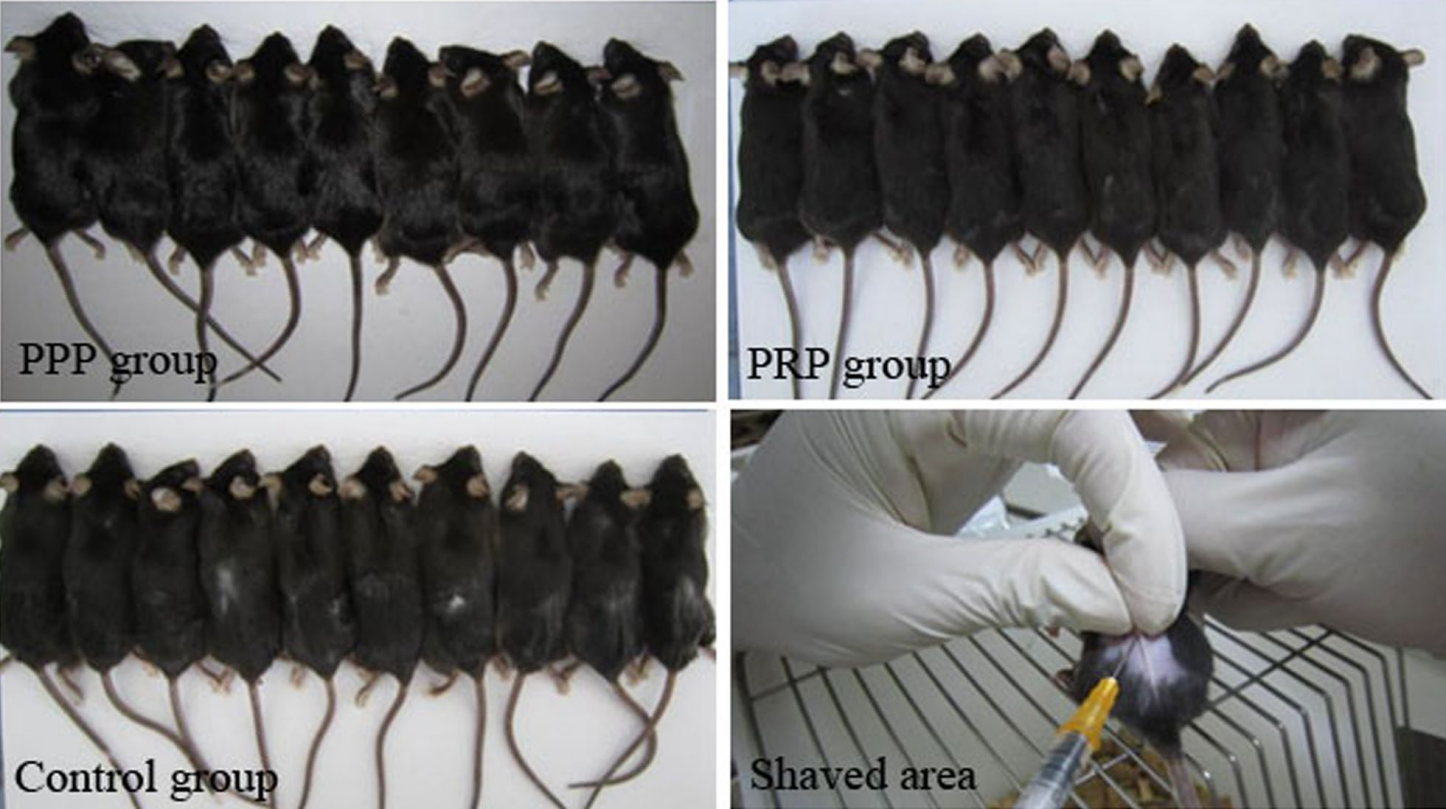
Effect of platelet-rich plasma (PRP) on hair growth of C57BL/6 mice at 18 days of the last injection of PRP, platelet-poor plasma (PPP), and saline (Control)

In addition, diffuse darkening of the dorsal skin was observed after 18 days in each group. Mice injected with activated PRP exhibited near-complete hair regrowth, whereas PPP- and saline-injected mice did not show too much hair regrowth (Fig. 2).

### PRP promotes formation of new vessels

**Fig. 3:**
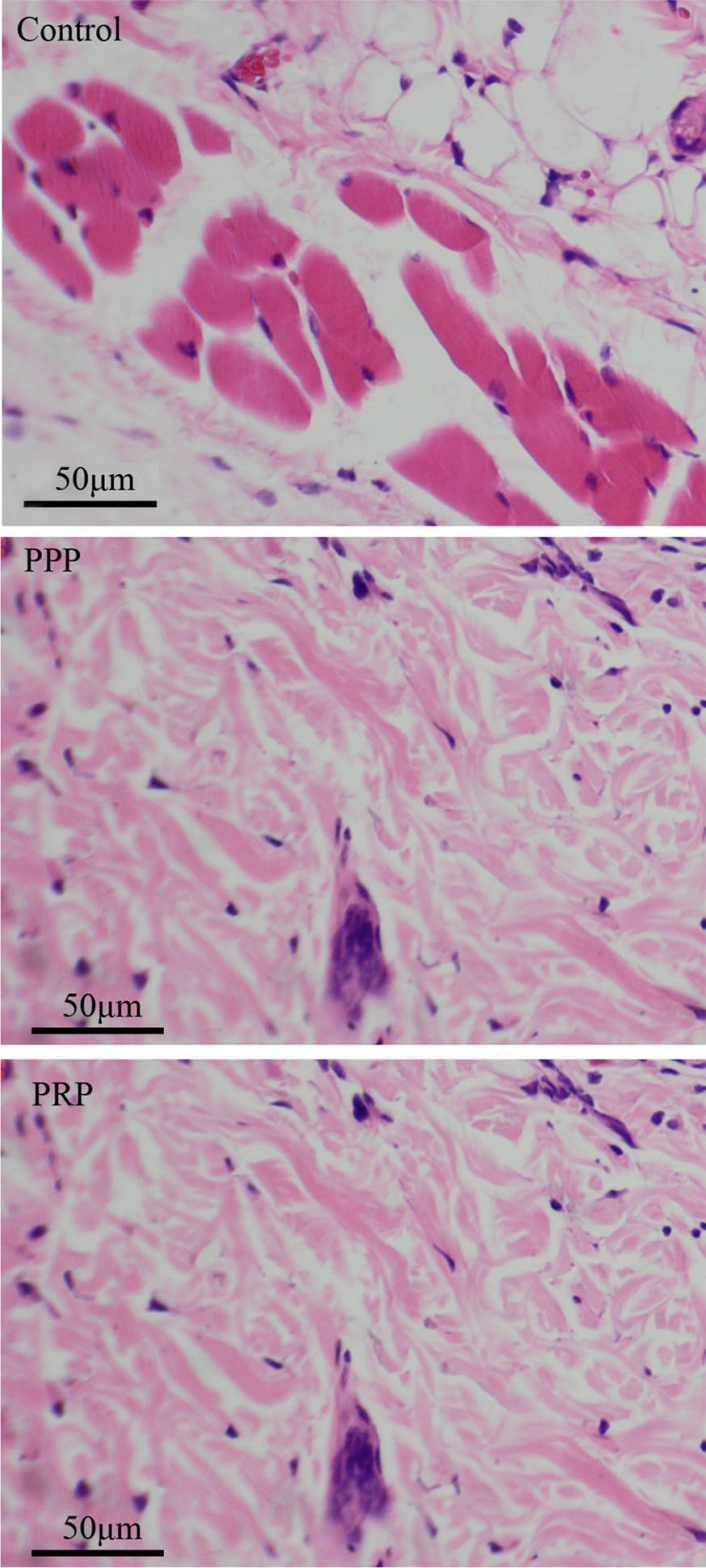
Histologic observation of the dorsal skins with hematoxylin and eosin staining in the control, platelet-poor plasma (PPP), and platelet-rich plasma (PRP) groups, original magnification × 250

HE staining of the histological observation (Fig. 3) showed that in the control and PPP group, hair follicle structures arranged in concentric circles in the mouse skin stratum corneum were found. The center of the hair follicle was eosinophilic keratosis substance, and fewer vascular tissues were found beneath the epidermis. In addition, abundant of new hair follicles and new blood vessels were observed in the PRP group.

**Fig. 4:**
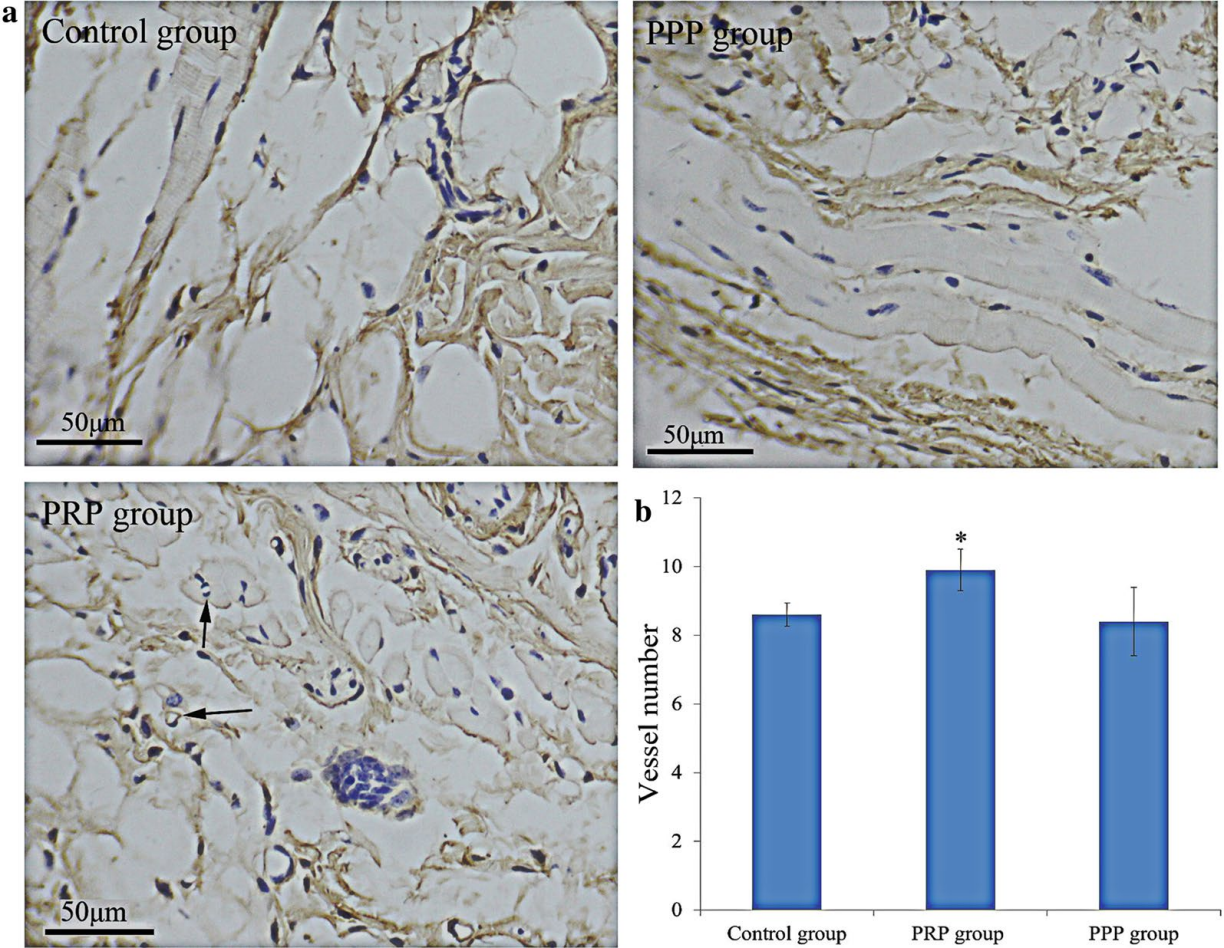
Effect of platelet-rich plasma (PRP) on angiogenesis by immunohistochemistry. **a** Positive vessels detected by immunohistochemical staining in the control, platelet-poor plasma (PPP), and PRP group, original magnification × 250. Arrows stand for positive vessels. **b** The relative number of vessels in the three groups. *p < 0.05 vs. control group

In order to confirm the effects of PRP on angiogenesis, we measured the expression of CD31 by immunohistochemistry in three different groups, and found that CD31 was mainly expressed in the vascular endothelial cells (Fig. 4a). Besides, the number of CD31-positive vessel in the PRP group (9.90 ± 0.60) was more than that in the control group (8.60 ± 2.34, *p* < 0.05, Fig. 4b). However, no significant difference of the number of CD31 vessel was found between the PPP group (8.40 ± 0.99) and the control group (8.60 ± 2.34, *p* > 0.05).

## Discussion

Recent reports showed that PRP had been widely applied in plastic surgery because of its ability to stop bleeding and promote wound healing [8, 9]. Since platelets damaged or rendered nonviable by PRP processing could not secrete bioactive growth factors [10], effective preparation methods were essential for obtaining high-quality PRP. The centrifugation of whole blood was the basic method used for producing PRP. However, the concentrations of PRP and growth factor widely varied using different commercial PRP separation systems based on centrifugation [11]. Furthermore, the natural variations in platelet concentration among individuals could also affect the final product of PRP [12]. Higher platelet concentrations of PRP were detrimental [13, 14], which might be attributed to the fact that some growth factors exerted an inhibitory effect on cell functions once a high enough concentration was reached [15, 16]. So the concentrations were measured after the preparation of PRP in our study. It has been reported that PRP is a concentration of platelets (3–5 fold the plasma baseline level) [17]. In this study, platelet concentration in PRP (6.5 × 10^9^ cells/ml) was seven times higher than that in whole blood (0.8 × 10^9^ cells/ml). Therefore, we adjusted the platelet concentration in PRP to 4 × 10^9^ cells/ml according to the baseline.

Several reports showed that the platelets in PRP were usually activated by thrombin with calcium chloride before use [6, 7]. The alpha granules of platelet began to secrete growth factors immediately once activated, and secreted almost all growth factors within an hour [18, 19]. Most PRP protocols in vitro experiments used bovine or autologous thrombin with or without calcium chloride to activate the platelets [8, 20], which was consistent with our study that PRP was activated by exogenous thrombin.

In addition, we found that the hair length in PRP group was longer than that in control group after 13 and 18 days of the last injection (*p* < 0.05). However, no significant difference in the hair length was found between the PPP group and the control group (*p* > 0.05). Furthermore, the number of CD31-positive vessel in PRP group was more than that in control group after 18 days of the last injection (*p* < 0.05). The reason may be that compared with PPP, PRP contains higher concentrations of various growth factors and has the ability to stimulate wound healing. Growth factors such as vascular endothelial growth factor (VEGF), platelet-derived growth factor (PDGF), and fibroblast growth factor (FGF) combined with PRP respective receptors play important roles in tissue angiogenesis and new organic structures growth [21–24]. PDGF and VEGF are closely associated with hair formation and follicle size [25, 26]. Various studies demonstrated that the use of PRP in hair transplantation could promote hair growth and accelerate larger follicular units [5, 7]. Together with earlier researches [6, 27], our study demonstrates that PRP could promote the hair growth.

A previous study found that the perifollicular vessel size changed with different stages of hair cycling, and VEGF was responsible for the angiogenesis associated with hair cycling [28]. So in present study, injected PRP might promote angiogenesis and hair growth by providing growth factor VEGF. In addition, platelets could induce differentiation of endothelial progenitor cells to mature endothelial cells [29], which might be another important evidence for promoting angiogenesis.

In the present study, as shown in Fig. 2, mice injected with activated PRP exhibited near-complete hair regrowth, whereas PPP- and saline-injected mice did not show too much hair regrowth, which meant that all the 3 groups had the hair regrowth but the PRP group had more significantly regrowth than the other two groups. The chronology of hair follicle cycling is as follows: catagen → telogen → anagen → catagen [30]. At 7 weeks of age, all hair follicles were in the telogen stage. The mice used in this study were 6 weeks old, and Fig. 2 was taken in 18 days after injection. The mice might be in anagen stage when Fig. 2 was taken. Therefore, it was not surprising that it showed little hair growth in the control group.

One limitation of our study is that the molecular mechanical properties of the role of PRP on hair growth were not be examined. The second limitation was that the plucked hair was randomly chosen by a person so the hair of the required or expected length might be chosen. The third limitation was that three independent fields (250 ×) were randomly selected to calculate the number of positive vessels in immunohistochemical analysis. It might be better to choose fields in association with the bulge region or the area close to a hair follicle as we referred that the increase in the number of vessels might be associated with hair growth. In addition, statistical analysis was not performed on the number of hair follicle among different groups.

## Conclusion

In conclusion, our data suggested that PRP might stimulate hair length growth and increase the number of hair follicles by inducing angiogenesis. Considering the limited evidence as to its clinical efficacy and safety, further studies are still needed to investigate the precise mechanism of PRP on hair growth.

## Abbreviations

PRP: platelet-rich plasma
PDGF: platelet-derived growth factor
VEGF: vascular endothelial growth factor
FGF: fibroblast growth factor
HGF: hepatocyte growth factor
TGF: transforming growth factor
PPP: platelet-poor plasma
SD: standard deviations
